# Addition of histamine to subcutaneously injected *Plasmodium berghei* sporozoites increases the parasite liver load and could facilitate whole-parasite vaccination

**DOI:** 10.1186/s12936-015-0552-3

**Published:** 2015-01-28

**Authors:** Johannes Pfeil, Jan F Heine, Ann-Kristin Mueller

**Affiliations:** Centre for Childhood and Adolescent Medicine (General Paediatrics), University Hospital, Im Neuenheimer Feld 430, 69120 Heidelberg, Germany; Centre for Infectious Diseases, Parasitology Unit, University Hospital, Heidelberg, Germany; German Centre for Infectious Diseases (DZIF), Heidelberg, Germany

**Keywords:** Histamine, Heparin, Sporozoite, SPZ, Chemoprophylaxis with sporozoites, Whole-parasite immunization, Malaria vaccine

## Abstract

**Background:**

Whole-parasite immunization remains the benchmark in malaria vaccine development. A major bottleneck in the translation of whole-parasite immunization towards routine vaccination is the mode of administration, since high degrees of protection are currently only achieved by intravenous, and not by intradermal or subcutaneous injection of viable parasites. It is known that only a small proportion of subcutaneously administered parasites reach the subsequent liver stage and low parasite liver load was shown to be associated with low protective efficacy. The objective of this analysis was to evaluate whether the liver load following subcutaneous parasite injection could be augmented by co-administration of pro-inflammatory or anti-coagulatory drugs.

**Methods:**

In the C57BL/6 *Plasmodium berghei* ANKA model, the clinical outcome (time to patent blood stage infection and survival) and relative parasite liver load was assessed in mice infected by subcutaneous or intramuscular sporozoite (SPZ) administration in the presence or absence of histamine and heparin supplementation in comparison to intravenously administered SPZ. In addition, a vaccination experiment was carried out to assess the protective efficacy of an improved, histamine-supplemented subcutaneous immunization regimen.

**Results:**

The parasite liver load following subcutaneous SPZ administration can be significantly increased by co-administration of histamine and heparin. A dose-dependent relation between parasite liver load and histamine dosage was observed. However, despite a relatively high parasite liver load, the protective efficacy of histamine-supplemented subcutaneous immunization remains inferior as compared to intravenous SPZ administration.

**Conclusions:**

Histamine supplementation might facilitate the future development of a non-intravenous whole-parasite vaccine. Further investigations are needed to reveal the effect of histamine supplementation and subcutaneous SPZ administration on the acquisition of protective immunity.

**Electronic supplementary material:**

The online version of this article (doi:10.1186/s12936-015-0552-3) contains supplementary material, which is available to authorized users.

## Background

For six decades, vaccination with whole attenuated parasites has remained the gold standard in malaria vaccine development and is the only way to ensure complete protection in vaccinated individuals. Following the first report of protective immunity in mice receiving irradiated sporozoites (SPZ) [1], the approach was transferred to human trials and initially SPZ were delivered by the bites of more than 1,000 irradiated infectious mosquitoes [2,3]. Since then, it has been shown that not only irradiation, but also other modes of attenuation, such as administration of protective drugs [4,5] and genetic modification [6] can be used. Whole-parasite malaria vaccination had been considered unfeasible for routine vaccination until recently when it returned to the focus of anti-malarial vaccine development following the development of aseptic, purified, vialed, and cryopreserved NF54 *Plasmodium falciparum* SPZ (PfSPZ vaccine) [7].

The initial clinical assessment of the irradiated PfSPZ vaccine reported limited immunogenicity and protection when administered intradermally (id) [8]. However, intravenous (iv) administration resulted in higher immunogenicity and improved protective efficacy in a subsequent study [9]. Complementary studies in the rodent model demonstrated that only a small number of id or subcutaneously (sc) injected parasites reach the intrahepatic stage. Moreover reduced parasitic liver load was associated with low protective efficacy following id SPZ administration [8,10].

Concerning the translation of whole-parasite immunization towards a routine malaria vaccine, highly protective non-iv immunization regimens would be preferred especially when the vaccine is intended for the use in paediatric populations. Thus, improving the infectivity of non-iv SPZ administration routes is an important objective for further development of whole-parasite vaccination.

In natural malaria infections, SPZ are transmitted to humans by the bite of infective female *Anopheles* mosquitoes. The bite elicits an acute inflammatory reaction at the feeding site, resulting from the saliva of mosquitoes that contains pro-inflammatory [11] as well as anti-hemostatic [12] agents that facilitate the blood feeding process. The hypothesis of this analysis is that the local inflammatory reaction may not only benefit the feeding mosquito but also improve the chances of parasites successfully entering the blood stream and in turn, establishing a malaria infection in the mammalian host.

The objective of this study was to investigate the infectivity of sc and intramuscular (im) SPZ administration in the presence or absence of both pro-inflammatory (histamine) and anti-haemostatic (heparin) drugs in the rodent C57BL/6 *Plasmodium berghei* (ANKA strain) model. In addition, the protective efficacy of histamine-supplemented sc whole-parasite immunization was evaluated.

## Methods

### Ethics statement

All animal experiments were performed according to FELASA category B and GV-SOLAS standard guidelines. Animal experiments were approved by German authorities (Regierungspräsidium Karlsruhe, Germany), § 8 Abs. 1 Tierschutzgesetz (TierSchG).

### Data assessment and statistical analysis

Data assessment and statistical analysis were performed using Stata IC/13.0 (StataCorp LP, College Station, TX, USA) and Prism Version 5.0b (GraphPad Software, San Diego California, USA). Log rank-test was used to compare survival distributions. Within groups of infected animals, Student’s t-test or Mann–Whitney-U-test were applied to compare parasite liver load detected by qRT-PCR or *in vivo* bioluminescence measurement, as appropriate. The clinical outcome of the final immunization experiment was assessed using Fisher’s exact test.

### Infection, immunization and challenge procedure

Freshly dissected *P. berghei* (ANKA strain) salivary gland SPZ were injected into C57BL/6 mice either sc in the neck skin fold, im in the thigh muscle or iv into the tail vein. SPZ were administered in PBS in a total volume of 100 μl, supplemented with 5 IU of heparin (Heparin 5000 IU/ml, Ratiopharm GmbH, 89079 Ulm, Germany) and varying amounts (1, 3 or 100 μg) of histamine (10 mg/ml histamine-dihydrochlorid, ALK-prick, ALK-Abell Arzneimittel GmbH, 22876 Wedel, Germany). For im administration, the volume was reduced to 50 μl.

For immunization under chloroquine (CQ) chemoprophylaxis (chloroquine chemoprophylaxis with sporozoites; CQ-CPS), mice were immunized by a prime-two boost regimen administered iv or sc at weekly intervals. CQ chemoprophylaxis was continuously supplied in the drinking water (CQ-DW) as previously described [13] with an elevated CQ concentration (300 mg/l instead of 288 mg/l). CQ-DW was introduced concomitant with the first (prime) SPZ administration and maintained until 14 days after the final (boost 2) immunization. Mice were challenged four weeks after withdrawal of CQ-DW by iv injection of 10^4^ freshly dissected *P. berghei* salivary gland SPZ.

### Parasitaemia read-out

Thin blood smears were obtained at days 4, 5, 7, 10, and 14 post-infection. Blood smears were Giemsa-stained and 25 light fields, each representing approximately 400 single-layer erythrocytes, were assessed by light microscopy. Malaria infection was reported if at least two infected red blood cells were detected within the same slide on at least one occasion.

### Quantification of parasite liver load by real-time PCR

For quantification of the parasite load in the liver by real-time qRT-PCR, C57BL/6 mice were infected with 10^4^ SPZ iv or sc, with or without supplementation of 5 IU heparin and 3 μg or 100 μg of histamine.

Mice were sacrificed at 42 or 48 hours post-infection, and livers were removed and homogenized. Total RNA was isolated with the RNeasy kit (Qiagen), and complementary DNA (cDNA) was synthesized with the RETROScript kit (Ambion), according to the manufacturer’s instructions. Real-time PCR was performed with the ABI 7500 sequence detection system and Power SYBR Green PCRMasterMix (Applied Biosystems), according to the manufacturer’s instructions, using gene-specific primers for the *P. berghei* 18SrRNA [GenInfo Identifier (GI), 160641] (forward: 5′-AAGCATTAAATAAAGCGAATACATCCTTAC-3′; reverse: 5′-GGAGATTGGTTTTGACGTTTATGTG-3′) and the mouse GAPDH gene (GI, 281199965) (forward: 5′-CGTCCCGTAGACAAAATGGT-3′; reverse:5′-TTGATGGCAACAATCTCCAC-3′). Temperature profile was as follows: 95°C for 10 min, followed by 40 cycles of 95°C for 15 sec, 55°C for 45 sec and 60°C for 1 min.

### *In vivo* quantification of parasite load

The transgenic *P. berghei* line 676m1cl1 (Pb GFP-Luc_con_) [14] was used for *in vivo* imaging. Luciferase activity was visualized using an *in vivo* imaging System (IVIS 100; Caliper Life Sciences, USA) as previously described [15]. In brief, animals were anesthetized using isofluorane, their belly was shaved and de-haired. D-luciferin (Synchem Laborgemeinschaft OHG, Germany) was dissolved in PBS and a total of 2.5 mg/mouse were injected intraperitoneally. Bioluminescence imaging was acquired with an exposure time of 180 sec directly following administration of D-luciferin and analysed using Living Image 2.50.1 (Xenogen Corp., Hopkinton, MA, USA).

## Results

### Infection by subcutaneous SPZ with histamine and heparin results in reduced prepatency and survival time

An initial experiment was conducted to assess the clinical outcome of mice infected by sc injection of 10^4^ SPZ, administered either in PBS (n = 10), or in PBS supplemented with 5 IU of heparin and 1 μg (1 μg sc, n = 10) or 3 μg (3 μg sc, n = 10) of histamine.

**Figure 1:**
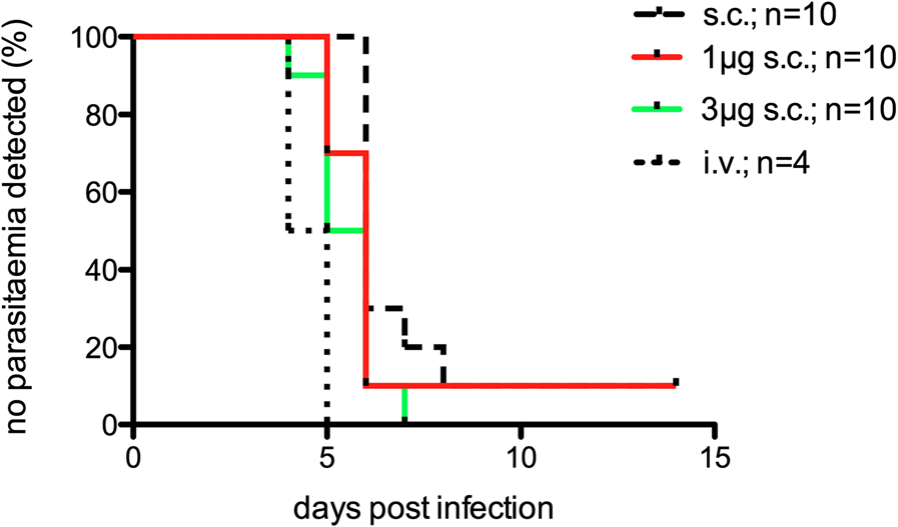
**Survival time of malaria-infected animals.** Animals were infected by a single injection of 10^4^ SPZ, administered either in PBS (n = 10), or in PBS supplemented with 5 IU of heparin and 1 μg (1 μg sc, n = 10) or 3 μg (3 μg sc, n = 10) of histamine.

**Figure 2:**
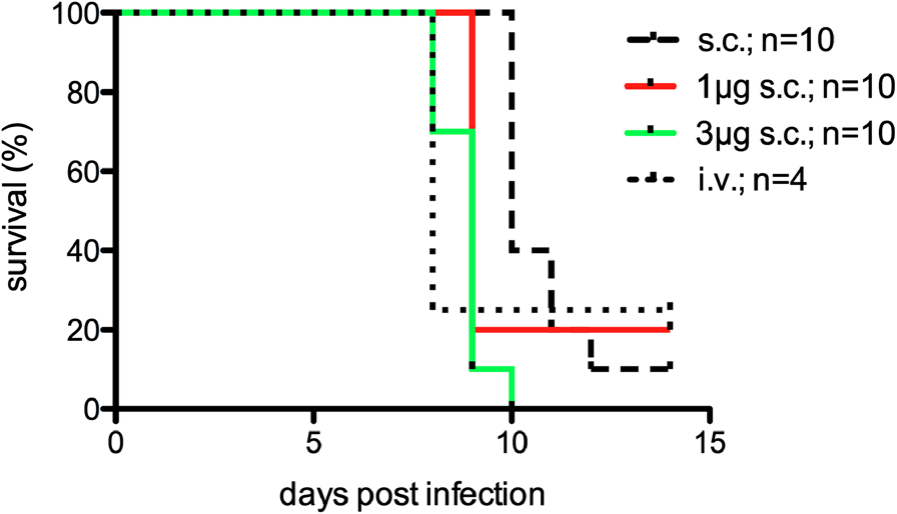
**Survival time of malaria-infected animals.** Animals were infected by a single injection of 10^4^ SPZ, administered either in PBS (n = 10), or in PBS supplemented with 5 IU of heparin and 1 μg (1 μg sc, n = 10) or 3 μg (3 μg sc, n = 10) of histamine.

The addition of histamine and heparin decreased the time period to first microscopic detection of parasites in the blood smear (prepatency), and this effect seemed to be dependent on the total dosage of histamine supplementation. Mean prepatency was 6.9 (95% CI 5.1-8.7), 6.5 (4.6-8.4) and 5.5 (4.9-6.1) days in the sc, 1 μg sc and 3 μg sc groups, respectively. In comparison to the sc group, prepatency was significantly shortened in mice that received SPZ together with 3 μg histamine (P <0.05), but not in those that received 1 μg histamine (P = 0.25). In all groups, the mean prepatency was longer than expected after iv infection (usually three to four days), which was confirmed by a small group of four control animals infected with 10^4^ SPZ iv (Figure 1). In addition, the survival time within these groups of SPZ-infected animals was observed. The mean survival time post-infection decreased from 10.8 (95% CI 9.9-11.7) to 10 (8.5-11.5) and 8.8 (8.3-9.3) days in the sc, 1 μg sc and 3 μg sc groups, respectively. The survival time was significantly shortened in animals receiving SPZ supplemented with 3 μg histamine *versus* sc controls (P <0.001), but not in the 1 μg sc *versus* sc group (P = 0.22, Figure 2).

### Histamine supplementation of subcutaneously injected SPZ increases the parasite liver burden

**Figure 3:**
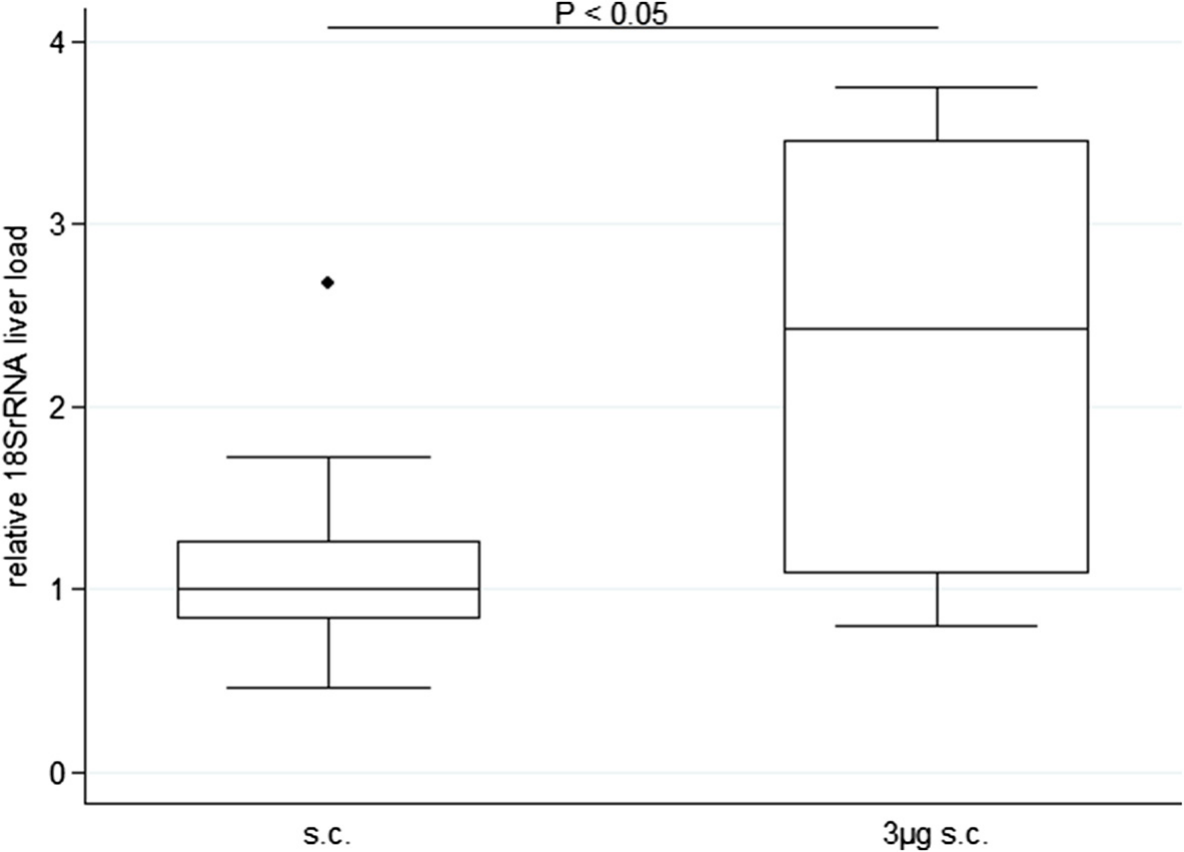
**Relative 18S rRNA liver load following a single injection of 10** ^**4**^ **sporozoites.** Animals were infected with SPZ sc in PBS plus 3 μg histamine and 5 IU heparin (3 μg his; n = 10) or in PBS only (sc; n = 9). Liver load was obtained at 42 hrs after infection.

**Figure 4:**
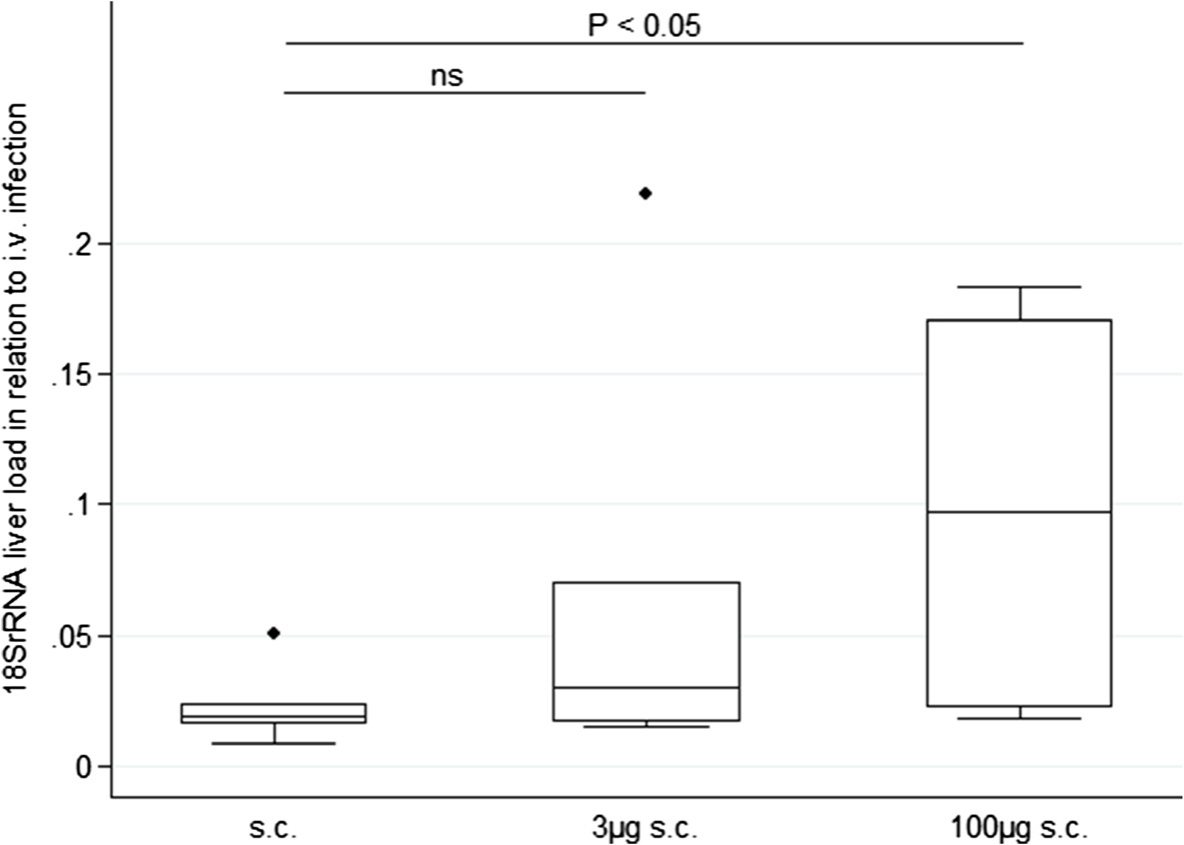
**Relative 18S rRNA liver load following subcutaneous infection in relation to intravenously infected control animals.** Animals were infected sc by a single injection of 10^4^ SPZ. SPZ were administered either without supplementation (sc, n = 7) or supplemented with 3 μg histamine and 5 IU heparin (3 μg sc; n = 7) or 100 μg histamine and 5 IU heparin (100 μg sc, n = 5). The liver load was obtained at 48 hrs after infection and is expressed in relation to the median 18S rRNA liver load of four iv-infected control mice.

Following this clinical observation, further experiments were conducted to evaluate whether parasitic liver burden as determined by both qRT-PCR and *in vivo* imaging were increased in animals infected with SPZ plus histamine compared to animals infected with SPZ in PBS only. In animals infected sc by 10^4^ SPZ in PBS supplemented with 3 μg histamine and 5 IU of heparin (3 μg sc; n = 9), the mean parasitic liver load was increased by approximately 2.5-fold (P <0.05) as compared to animals infected with 10^4^ SPZ sc in PBS (sc; n = 11). A wide inter-individual variance in parasite liver burden was noted (Figure 3). Based on the clinical outcome described above, a dose-dependent effect of histamine supplementation on the resulting parasite liver load was assumed. According to this hypothesis, further experiments compared the parasite liver load of mice infected subcutaneous with 10^4^ SPZ in PBS (sc, n = 7), or in PBS supplemented with 5 IU of heparin and either 3 μg (3 μg sc; n = 7) or an increased dosage of 100 μg (100 μg sc; n = 5) of histamine. The parasite liver load was calculated by the transcriptional abundance of 18SrRNA versus the GAPDH reference. Four control mice were infected at the same time point with equivalent doses of SPZ iv, and the liver load of sc infected mice was expressed in relation to the median liver load of these iv-infected control animals. The median relative liver burden detected was 2, 3 and 10% in the sc, 3 μg sc and 100 μg sc groups, respectively, and subject to wide inter-individual variance (Figure 4).

**Figure 5:**
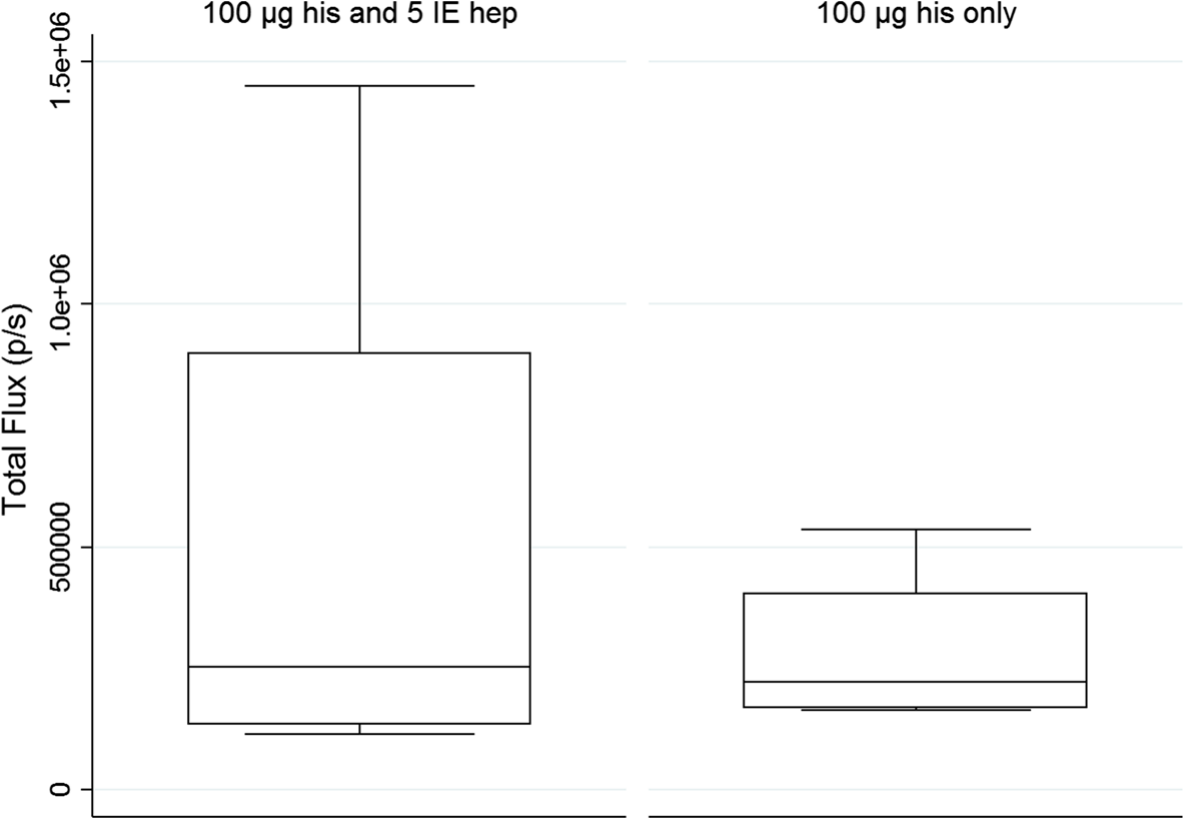
***In vivo*** **liver load following intramuscular in the presence or absence of heparin supplementation.** Animals were infected by a single injection of 10^4^ luciferase-expressing SPZ im supplemented with 100 μg histamine with (n = 4) or without (n = 4) 5 IU heparin. No significant difference between heparin supplemented and non-supplemented mice was detected.

**Figure 6:**
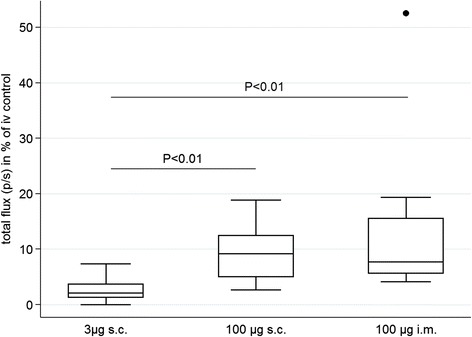
***In vivo*** **liver load in relation to intravenously infected control animals.** Mice were infected with 10^4^ SPZ supplemented with either 3 μg histamine and 5 IU heparin sc (3 μg s.; n = 8) or 100 μg histamine and 5 IU heparin sc (100 μg sc; n = 8) or 100 μg histamine with (n = 4) or without (n = 4) 5 IU heparin im (mice are displayed combined in group 100 μg im; total n = 8). The intrahepatic flux was obtained at 48 hrs post-infection and is expressed in relation to the median flux measurement of four iv-infected control mice.

To validate the results obtained from qRT-PCR analysis, *in vivo* imaging was applied as a second read-out for parasite liver development. Intrahepatic flux was measured in mice infected sc with 10^4^ luciferase-expressing SPZ plus 5 IU of heparin and either 3 μg (3 μg sc; n = 8) or 100 μg (100 μg sc; n = 8) histamine. In addition, a group of mice receiving intramuscular injections of 10^4^ luciferase-expressing SPZ and 100 μg histamine with (n = 4) or without (n = 4) 5 IU of heparin was included in the in vivo experiments. In this small experiment, the addition or absence of heparin did not influence the parasite liver load of im infected mice (Figure 5), and all animals were thus summarized in a single group (100 μg im). As before, the intrahepatic flux was expressed in relation to the median of four simultaneously iv-infected control animals. The median relative parasite liver load was 2, 9 and 7% for the sc 3 μg, sc 100 μg and im 100 μg group, respectively (Figure 6).

### The parasite liver load following sc injection is scalable by injection of increasing SPZ numbers

In preparation of subsequent immunization experiments, it was assessed if a parasite liver load comparable to iv SPZ administration could be achieved by combining histamine supplementation with increasing numbers of sc-injected SPZ.

Based on the previously determined relative parasite liver load achieved by sc *versus* iv infection, two independent experiments were designed to compare: A) the parasite liver load in mice infected sc with 2*10^5^ SPZ plus 3 μg of histamine and 5 IU of heparin versus mice infected with 10^4^ SPZ iv; and B) the parasite liver load in mice infected sc with 3*10^4^ SPZ plus 100 μg of histamine and 5 IU of heparin versus mice infected with 10^3^ SPZ iv. *In vivo* measurement at 48 hrs post-infection demonstrated that the mean parasite liver burden in sc-infected mice reached at least 50% of iv-infected control animals (Additional file 1 Figures S1a and S1b).

### Despite high parasite liver load, the protective capacity of histamine-supplemented sc CQ-CPS remains inferior to iv CQ-CPS

An immunization experiment was conducted to assess the protective efficacy of whole parasite immunization delivered by improved sc SPZ administration *versus* conventional iv immunization. Groups of animals were infected with a prime-two boost regimen of 3*10^4^ SPZ under CQ chemoprophylaxis either sc in PBS (CQ-CPS sc; n = 6), with 3*10^4^ SPZ sc in PBS supplemented with 5 IU heparin and 100 μg histamine (CQ-CPS 100 μg sc; n = 8) or by 3*10^3^ SPZ in PBS iv (CQ-CPS iv; n = 8). Following a challenge with 10^4^ SPZ iv, it was observed that all previously iv immunized animals remained completely protected from blood-stage infection, while six out of eight animals in the CQ-CPS 100 μg sc group and all animals in the CQ-CPS sc group developed blood-stage infection. Thus, the rate of protected animals in the CQ-CPS 100 μg sc and the CQ-CPS sc groups remained significantly lower compared to the iv control group (both P <0.01), while the difference between both sc immunized groups was non-significant (P = 0.47; Table 1). Despite the low protective capacity of sc immunization against blood-stage infection, immunized animals were still protected from severe malaria disease as none of the immunized animals succumbed or developed signs of severe neurological disease (defined by a rapid murine coma and behaviour scale <5) [16] until day 14 post-challenge (Table 1).

**Table 1:** **Protection after chloroquine chemoprophylaxis with sporozoites immunization**

	**Week**	**CQ control**	**CQ-CPS iv**	**CQ-CPS 100 μg sc**	**CQ-CPS sc**
		**n = 4**	**n = 8**	**n = 8**	**n = 8**
Immunization/drug treatment (prime; boost 1; boost 2)	0; 1; 2	- / CQ-DW	3 × 10^3^ SPZ iv / CQ-DW	3 × 10^4^ SPZ + 100 μg his and 5IU hep sc / CQ-DW	3 × 10^4^ SPZ sc / CQ-DW
Challenge	8	10^4^ SPZ iv	10^4^ SPZ iv	10^4^ SPZ iv	10^4^ SPZ iv
Animals protected		0/4	8/8	2/8	0/6*
Animals surviving by day 14 post-challenge		1/4	8/8	8/8	6/6

*CQ*-*DW*: chloroquine supplemented drinking water; his: histamine; hep: heparin.

*CQ*-*DW* was maintained in all animals from prime immunization until 2 weeks after the final boost immunization.

*two animals died during the immunization process for unknown reasons.

## Discussion

This study in the rodent malarial model demonstrates that the parasite liver load following sc or im SPZ injection can be increased by co-administration of heparin and histamine. The increase in parasite liver burden correlates with histamine supplementation in a dose-dependent manner, while heparin supplementation might be dispensable. Parasite liver load is thought to be associated with protective efficacy achieved by whole-parasite immunization [8,10]. Therefore, the concept of histamine supplementation might represent an important contribution to the ongoing efforts in the development of an efficacious non-iv whole-parasite vaccine.

Within the analyses, it was found that supplementation with 100 μg histamine and 5 IE heparin increases the parasite liver burden by approximately five-fold as compared to conventional sc SPZ administration. The parasite liver load thereby reaches approximately 10% of the level achieved by iv infection, which is superior to any previous report on im, sc or id SPZ administration [17].

Importantly, sc histamine administration is not restricted to the rodent model but has previously been applied as an adjuvant to immunotherapy in oncologic phase II and III clinical trials. Subcutaneous administration of 1 mg histamine-dihydrochloride was found to be safe [18], even when applied in an outpatient setting [19]. A safety assessment in rats demonstrated that repeated doses of 500 mg/kg or higher elicited acute tissue damage, while the repeated injection of up to 100 mg/kg did not result in relevant side effects besides a local inflammatory reaction [20]. Thus, the concept of histamine co-administration is likely to be safe and feasible not only in the rodent model but also in human whole-parasite vaccination.

The increase in parasitic liver obtained through histamine co-administration has to be interpreted with caution as it might be explained by two different mechanisms. Firstly, the local inflammation and vasodilation induced by histamine injection may increase the number of SPZ entering a capillary and thus increase the total number of parasites reaching the liver stage. It is known that the majority of id administered SPZ remain in the skin for hours before entering the bloodstream and invading a hepatocyte, whereas iv-injected SPZ reach the liver within minutes [21]. Thus, it cannot be excluded that co-administration of histamine enables SPZ to enter the blood stream at an earlier time point rather than in greater number. In this case, the increasing parasite liver load detected at a single time point post-infection might not, or only in parts, represent an increase in total liver load over time but rather a shift of the maximum liver burden to earlier time points post-infection. For the same reason, determination of the parasite liver load obtained by sc *versus* iv administration at a single time point post-infection represents a minimal and thus conservative estimate of the real total liver load obtained by sc injection. While it would be desirable to determine parasite liver burden over time, such measurements remains technically challenging as *in vivo* detection of intrahepatic liver development at later time points is masked by increasing blood-stage infection.

A previous study attempted to enhance the protective immunogenicity of injections either by topical application of imiquimod or by local ‘tape-stripping’ of the skin at the infection site. Interestingly, mice immunized by SPZ injected at a tape-stripped skin site demonstrated superior protection against a subsequent "by bite" SPZ challenge [22]. Tape-stripping of the injection is likely to induce local inflammation, and the superior protection reported might indicate that local inflammation increases the number of SPZ reaching the liver and thereby augments the total parasite liver load.

In a CPS vaccination experiment, the protective efficacy of a prime-two boost sc immunization regimen supplemented with 100 μg histamine and 5 IU heparin was compared *versus* a non-supplemented sc control group and an iv control group immunized by a ten-fold lower SPZ dosage. Despite the earlier observation of at least 50% parasite liver burden in histamine-supplemented sc *versus* iv immunized animals, the protection achieved by histamine-supplemented sc immunization remained inferior in comparison to the iv control group and was at best slightly higher than in the non-supplemented sc immunized controls. While it cannot completely be excluded that a slightly lower parasite liver load might sufficiently explain this inferior protection, additional factors might contribute to the decreased efficacy of sc CQ-CPS immunization. It is possible that a high number of SPZ remaining in the animals’ tissue enables the development of invasion-blocking antibodies during the prime immunization, thus leading to a decreased parasite liver load in subsequent immunizations [10]. Alternatively, immune responses induced by SPZ remaining in the skin or draining lymph nodes [23] or a histamine-triggered increasing activity of myeloid suppressor cells [24] might impede the development of protective cellular intrahepatic immune responses. The specific reasons for the low protection induced by repeated sc immunization are an important question for future investigations. Additional approaches and measures like e.g. intradermal SPZ administration [22], SPZ administration at lower volumes [21] or by microneedle patches [25], or the use of radiation attenuated SPZ rather than CPS might be combined with the concept of histamine supplementation to develop a highly protective non-iv SPZ immunization regimen.

In the meantime, the current data suggests that even though sc immunization was insufficient to protect against blood-stage infection, it still seems to prevent the onset of experimental cerebral malaria. This observation could be explained by blood-stage directed immune responses induced by CPS [26] and might represent an additional benefit obtained by CPS immunization. In summary, histamine supplementation represents a novel and conceptually innovative approach that could contribute to the future development of a non-iv whole-parasite malaria vaccines.

## Conclusions

Histamine supplementation of sc-injected SPZ increases the parasite liver load in a dose-dependent manner. This novel concept might contribute to the future development of an efficacious non-iv whole-parasite vaccine. Further investigation is required to reveal the underlying causes of inferior protective efficacy obtained by sc immunization in comparison to the iv gold standard.

## Additional file

Additional file 1:
**Enhancement of parasitic liver load by increased numbers of injected sporozoites.** In two independent experiments, the *in vivo* parasitic liver load at 48 hrs after infection was assessed in A) mice infected sc with 2*10^5^ SPZ supplemented with 3 μg histamine and 5 IU of heparin (2*10^5^ + 3 μg sc; n = 4) *versus* control mice infected iv with 104 SPZ (10^4^ iv; n = 4) and B) in mice infected with 3*10^4^ SPZ supplemented with 3 μg histamine and 5 IU of heparin sc (3*10^4^ + 100 μg sc; n = 3) *versus* control mice infected iv with 3*10^3^ SPZ iv (3*10^3^ iv; n = 3). Large intra-individual differences in the intrahepatic parasite burden were observed, without significant differences between sc and iv-infected animals in experiment **A)** while the liver load of sc-infected animals remained approximately half of the iv control group in experiment **B)**.

## Abbreviations

CPS: Chemoprophylaxis with sporozoites
CQ: Chloroquine
CQ-CPS: Chloroquine chemoprophylaxis with sporozoites
CQ-DW: Chloroquine supplemented drinking water
Id: Intradermal
Im: Intramuscular
Iv: Intravenous
Sc: Subcutaneous
SPZ: Sporozoites
