# Supplementary figures and images for: Addition of histamine to subcutaneously injected Plasmodium berghei sporozoites increases the parasite liver load and could facilitate whole-parasite vaccination
Source: Malar J. 2015 Jan 28;14:36. doi: 10.1186/s12936-015-0552-3 (PMC4318155; doi:10.1186/s12936-015-0552-3)

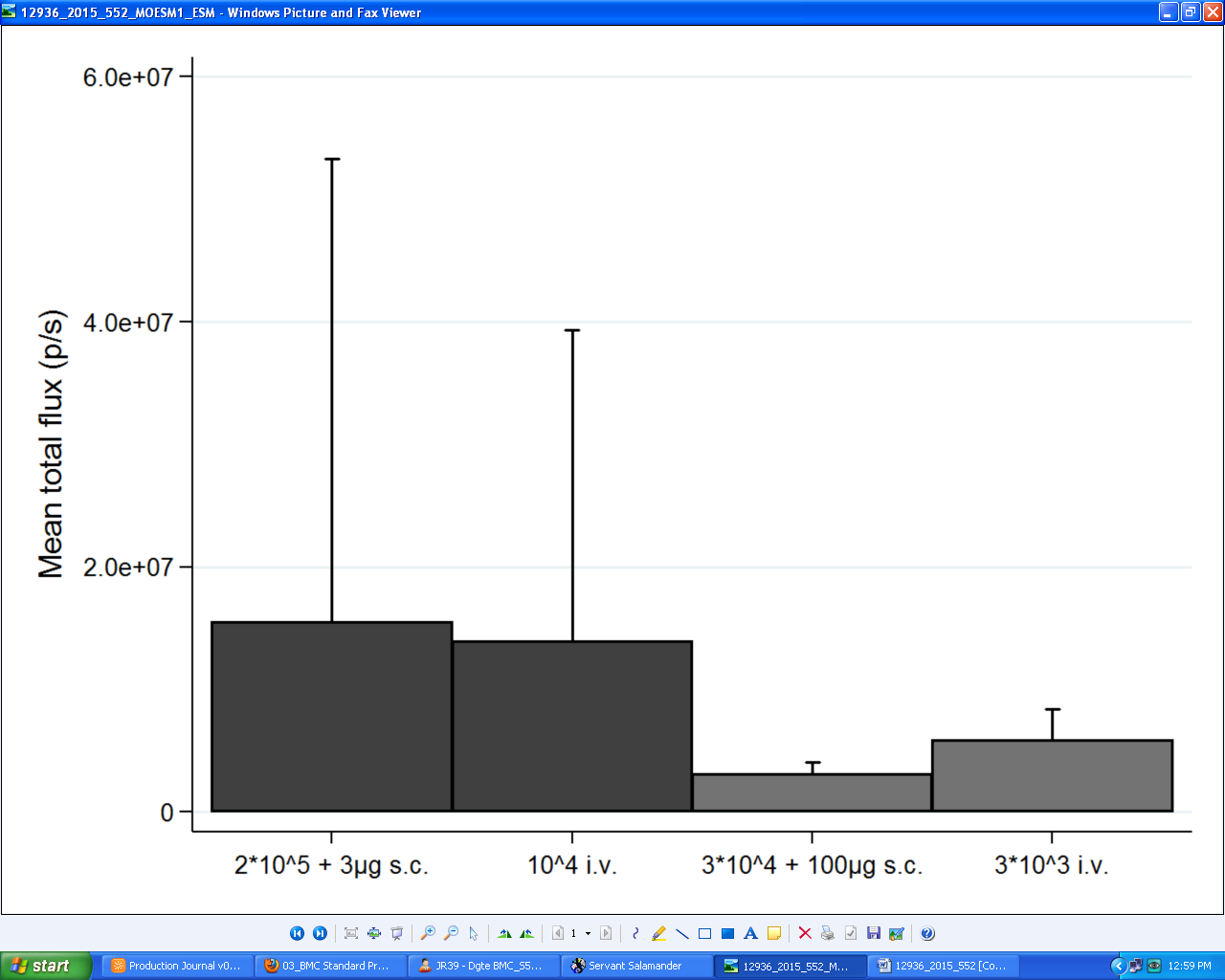


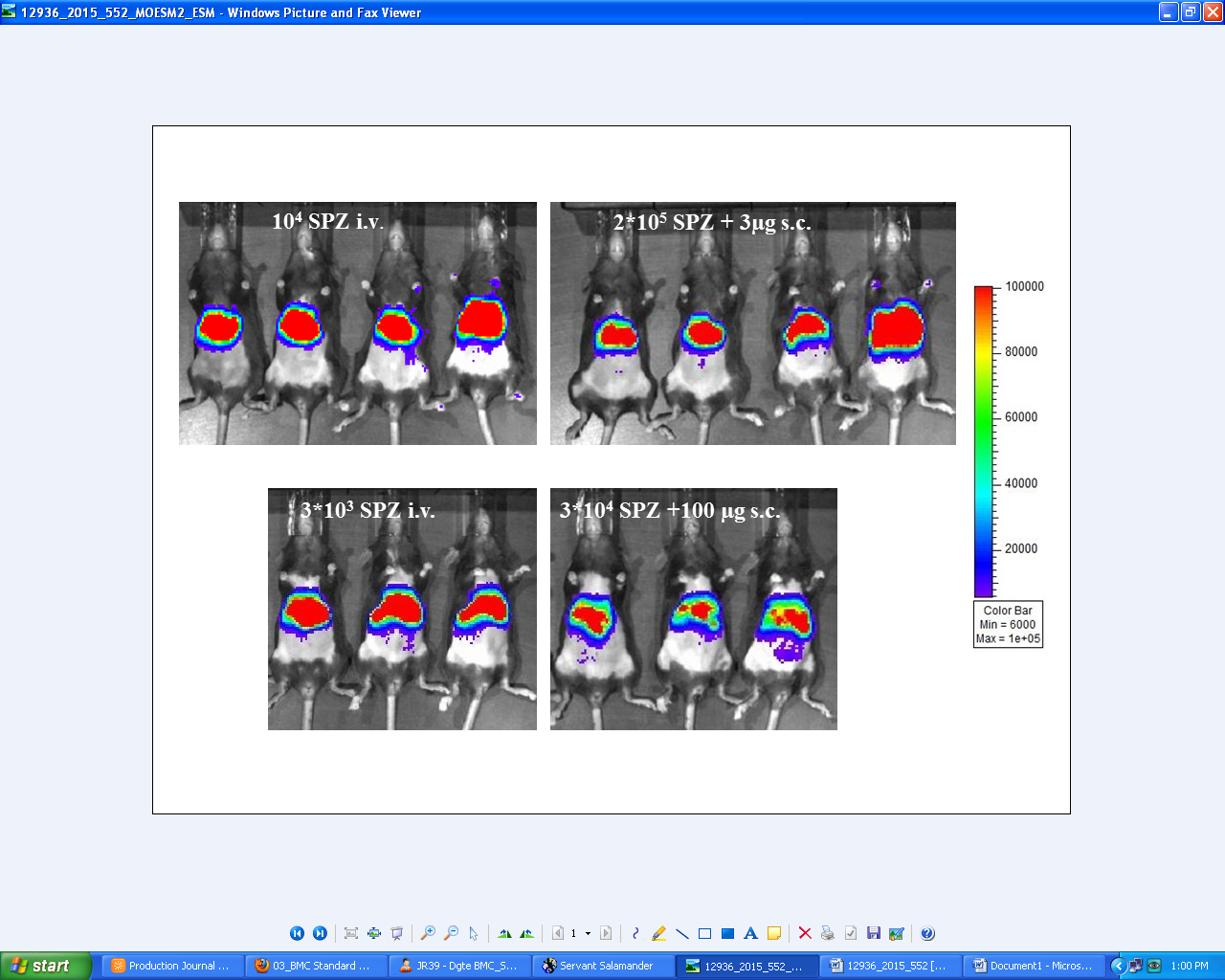

Supplement: Additional file 1: — Enhancement of parasitic liver load by increased numbers of injected sporozoites. In two independent experiments, the in vivo parasitic liver load at 48 hrs after infection was assessed in A) mice infected sc with 2*105 SPZ supplemented with 3 μg histamine and 5 IU of heparin (2*105 + 3 μg sc; n = 4) versus control mice infected iv with 104 SPZ (104 iv; n = 4) and B) in mice infected with 3*104 SPZ supplemented with 3 μg histamine and 5 IU of heparin sc (3*104 + 100 μg sc; n = 3) versus control mice infected iv with 3*103 SPZ iv (3*103 iv; n = 3). Large intra-individual differences in the intrahepatic parasite burden were observed, without significant differences between sc and iv-infected animals in experiment A) while the liver load of sc-infected animals remained approximately half of the iv control group in experiment B). [file 12936_2015_552_MOESM1_ESM.doc]
